# CRISPR/Cas9 genome editing clinical trials for neurodevelopmental disorders

**DOI:** 10.1192/j.eurpsy.2024.1216

**Published:** 2024-08-27

**Authors:** B. Abdelmoula, N. Bouayed Abdelmoula

**Affiliations:** Genomics of Signalopathies at the service of Precision Medicine -LR23ES07, Medical University of Sfax, Sfax, Tunisia

## Abstract

**Introduction:**

Recently, the new therapeutic approach based on genome editing using the CRISPR/Cas9 system has been applied to treat cancer and other monogenetic disorders. CRISPR/Cas9 allows specific correction of the altered gene without affecting the rest of the genome.

**Objectives:**

The aim of this study was to report the current CRISPR/Cas9 genome editing clinical trials in neurodevelopmental and mental disorders.

**Methods:**

We conducted a search via the ClinicalTrials platform to describe clinical trials that have been conducted using the CRISPR/Cas9 genome-editing tool in neurodevelopmental disorders.

**Results:**

Our research revealed three clinical trials that used the CRISPR/Cas9 tool for diagnostic and therapeutic purposes. The first study aimed to investigate the pathological role of KMT2D mutations in 40 Kabuki syndrome patients in order to facilitate the identification and characterization of therapeutic strategies to improve symptoms, to identify the consequences of KMT2D mutations on epigenetic marker changes and cellular structural changes and to finally attempt gene correction by CRISPR/Cas9. The therapeutic approach was an epigenome editing approach aimed at increasing the expression of the wild-type KMT2D allele to restore the functional activity of a histone H3-lysine 4 (H3K4)-methyltransferase (MLL4) in treated mesenchymal stem cells. The second clinical trial aimed to validate gene editing based on CRISPR/Cas9 technology combined with AAV delivery for the correction of the most common MECP2 mutations in Rett syndrome both in vitro and in vivo. The third GENEPI clinical trial aimed to identify acetylation profiles as epigenetic markers to assess the causality of CREBBP and EP300 variants in Rubinstein-Taybi syndrome, which is considered as a genetic model of neurodevelopmental abnormality with an epigenetic component.

**Conclusions:**

CRISPR/Cas9 clinical trials in polygenic conditions, such as psychiatric disorders, could be envisaged at the level of the epigenetic component of these pathologies. This therapy could be applied ex vivo to perform tissue-specific gene editing.

**Disclosure of Interest:**

None Declared

